# DNA methylation patterns at and beyond the histological margin of early-stage invasive lung adenocarcinoma radiologically manifested as pure ground-glass opacity

**DOI:** 10.1186/s13148-021-01140-3

**Published:** 2021-08-19

**Authors:** Ziqi Jia, Yadong Wang, Jianchao Xue, Xiaoying Yang, Zhongxing Bing, Chao Guo, Chao Gao, Zhenhuan Tian, Zhenzhen Zhang, Hualei Kong, Qiye He, Zhixi Su, Yiying Liu, Yang Song, Dianjing Liang, Naixin Liang, Shanqing Li, Yuan Gao

**Affiliations:** 1 Department of Thoracic Surgery, Peking Union Medical College Hospital, Peking Union Medical College and Chinese Academy of Medical Sciences, Beijing, 100730 China; 2 Peking Union Medical College, Chinese Academy of Medical Sciences, Beijing, 100730 China; 3 Singlera Genomics Inc., Shanghai, 201318 China; 4 Institute of Physics, Humboldt University of Berlin, 12489 Berlin, Germany

**Keywords:** Lung adenocarcinoma, Surgical margin, DNA methylation, Ground-glass opacity

## Abstract

**Background:**

Early-stage lung cancers radiologically manifested as ground-glass opacities (GGOs) have been increasingly identified, among which pure GGO (pGGO) has a good prognosis after local resection. However, the optimal surgical margin is still under debate. Precancerous lesions exist in tumor-adjacent tissues beyond the histological margin. However, potential precancerous epigenetic variation patterns beyond the histological margin of pGGO are yet to be discovered and described.

**Results:**

A genome-wide high-resolution DNA methylation analysis was performed on samples collected from 15 pGGO at tumor core (TC), tumor edge (TE), para-tumor tissues at the 5 mm, 10 mm, 15 mm, 20 mm beyond the tumor, and peripheral normal (PN) tissue. TC and TE were tested with the same genetic alterations, which were also observed in histologically normal tissue at 5 mm in two patients with lower mutation allele frequency. According to the difference of methylation profiles between PN samples, 2284 methylation haplotype blocks (MHBs), 1657 differentially methylated CpG sites (DMCs), and 713 differentially methylated regions (DMRs) were identified using reduced representation bisulfite sequencing (RRBS). Two different patterns of methylation markers were observed: Steep (S) markers sharply changed at 5 mm beyond the histological margin, and Gradual (G) markers changed gradually from TC to PN. S markers composed 86.2% of the tumor-related methylation markers, and G markers composed the other 13.8%. S-marker-associated genes enriched in GO terms that were related to the hallmarks of cancer, and G-markers-associated genes enriched in pathways of stem cell pluripotency and transcriptional misregulation in cancer. Significant difference in DNA methylation score was observed between peripheral normal tissue and tumor-adjacent tissues 5 mm further from the histological margin (*p* < 0.001 in MHB markers). DNA methylation score at and beyond 10 mm from histological margin is not significantly different from peripheral normal tissues (*p* > 0.05 in all markers).

**Conclusions:**

According to the methylation pattern observed in our study, it was implied that methylation alterations were not significantly different between tissues at or beyond P10 and distal normal tissues. This finding explained for the excellent prognosis from radical resections with surgical margins of more than 15 mm. The inclusion of epigenetic characteristics into surgical margin analysis may yield a more sensitive and accurate assessment of remnant cancerous and precancerous cells in the surgical margins.

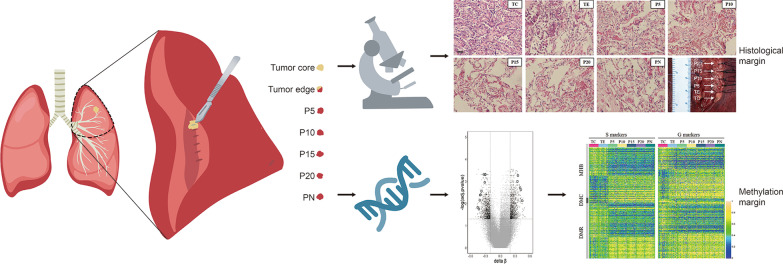

**Supplementary Information:**

The online version contains supplementary material available at 10.1186/s13148-021-01140-3.

## Background

Lung cancer continues to be the leading cause of cancer-related mortality [1, 2]. Early detection with low-dose computed tomography (LDCT) is widely applied and regarded as effective in reducing lung cancer mortality [3, 4]. Also, since 2020, low-dose high-resolution computed tomography (HRCT) has been widely applied in the screening of COVID-19 [5]. Thus, more patients accidentally found ground-glass opacities (GGOs) on CT scans, and some of them were diagnosed with early-stage lung cancers. GGO is a subjective description of a nodule or mass with a hazy feature of elevated density on computed tomography (CT) [6, 7]. GGO has mainly two types: nonsolid nodules, also known as pure GGO (pGGO), and partial-solid nodules with solid components observed on the mediastinal window [8]. Pure GGOs are constituted with benign and adenocarcinomas with noninvasive and invasive natures. Persistent pGGOs with large diameters and certain radiological features indicate progressive invasive lesions [9], which need surgical intervention [10]. The post-radical-surgical prognosis is excellent for pGGO, with 5-year disease-free survival of 100% [11, 12]. For the utmost preservation of lung function, surgical options and safe margin for sublobar resection are under discussion. Pathology research demonstrated that 20 mm was a safe margin [13], whereas segmentectomy at a distance of 15 mm was recommended after survival analyses [14].

Tumor-surrounding tissues frequently appear pathologically and histologically normal but contain precancerous lesions. Such phenomena are termed “field effect” or “field cancerization” [15]. It is considered surgically necessary to remove precancerous tissues to reduce local recurrence rate [16]. Field cancerization is driven by the accumulation of tumor-associated genetic and epigenetic alterations, such as DNA methylation [17–19]. Previous studies found that cancer-associated mutations in histologically negative margins, which had emerged long before the morphological alteration, could serve as “molecular margins” in the assessment of surgical margins of lung cancer [20]. Potential pre-morphological epigenetic changes beyond the histological margin of a tumor are yet to be discovered and described.

This study aimed to depict genome-wide high-resolution DNA methylation patterns at and beyond the histological margin of nodules in early-stage invasive lung adenocarcinomas (LADC) that appeared as pGGO. Moreover, we aimed to explore methylation markers indicative of a safe margin for pGGO resection. Samples were collected from the tumor core, tumor edge (the histological margin of tumor), distant normal tissue, and four surrounding tissues at increasing distances from their respective tumor core. We analyzed the DNA methylation profiles of those tissues to identify methylation markers significantly different between tumor core and peripheral normal tissue and revealed their landscapes in the surrounding tissues concerning their distance from the tumor core.

## Results

### Clinical characteristics and genetic profiles

Fifteen early-stage LADC patients were enrolled in the study. The clinical characteristics of these patients is listed in Table 1. All of the surgical samples were diagnosed as invasive adenocarcinoma with various pathological subtypes. The average age of the cohort was 52 years old. Eleven out of 15 patients were female, and only one patient had a smoking history. All of the lesions were characterized as pGGO on CT, with an average diameter of 1.51 cm (cm). Nine of them harbored an *EGFR* mutation, one patient harbored an *EML4-ALK* fusion, and one patient harbored an *ERBB2* exon 20 insertion. Nine patients who provided sufficient para-tumor tissue for DNA variation sequencing had the same DNA variations in TC and TE samples. *EGFR* mutations were identified in P5 of two patients with mutation allele frequency (MAF) lower than TC and TE. None of the DNA alterations were identified in other para-tumor tissues further from P5 (Additional file 1).

**Table 1:** Characteristics of patients with lung adenocarcinoma involved

ID	Age (y)	Sex	Smoking history	TNM	Pathology	Predominant pathological subtype	Size (cm)	Gene status
1	39	M	No	T_1a_N_0_M_0_	IA	Solid, acinar	0.75	*EGFR* L858R
2	52	M	Yes	T_1c_N_0_M_0_	IA	Acinar	2.2	*EGFR* G719A
3	60	F	No	T_1b_N_0_M_0_	IA	Leptic	2	*EGFR* L858R
4	55	F	No	T_1c_N_1_M_0_	IA	Solid, acinar	2.1	*EGFR* L858R
5	53	F	No	T_1b_N_0_M_0_	IA	Acinar, leptic	1.74	*EGFR* L858R, PTEN C136Y
6	55	F	No	T_1b_N_0_M_0_	IA	Papillary	1.1	Wild type
7	54	F	No	T_1a_N_0_M_0_	IA	Acinar, leptic	1	*EGFR* L858R
8	49	M	No	T_1b_N_0_M_0_	IA	Papillary, acinar, leptic	2	*EGFR* exon19del
9	72	F	No	T_1b_N_0_M_0_	IA	Acinar, leptic	1.6	*EGFR* L858R
10	31	F	No	T_1a_N_0_M_0_	IA	Acinar, leptic	1	*EML4*-*ALK* fusion
11	70	M	No	T_1a_N_0_M_0_	IA	Acinar	1.33	Wild type
12	68	F	No	T_1c_N_0_M_0_	IA	Acinar	2.7	not available
13	31	F	No	T_1b_N_0_M_0_	IA	Acinar	1.3	*ERBB2* exon20 ins
14	44	F	No	T_1a_N_0_M_0_	IA	Leptic	0.8	Wild type
15	49	F	No	T_1a_N_0_M	IA	Leptic	1	*EGFR *exon19del

### Quality control of DNA methylation data

**Fig. 1:**
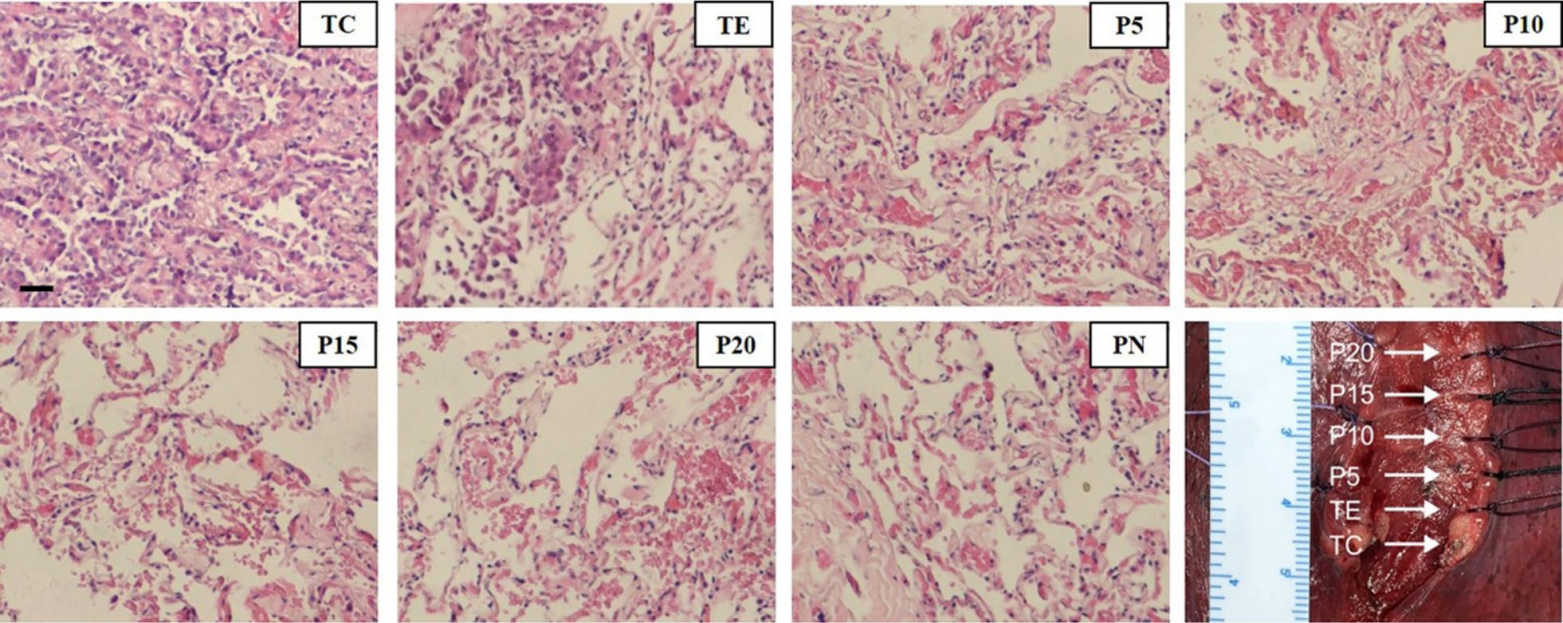
Light microscopic views with hematoxylin and eosin staining and macroscopic view of the samples of a representative patient. Samples include tumor core (TC), tumor edge (TE), peripheral normal tissue (PN), four surrounding tissues that were of increasing distance by 5 mm from their respective tumor core. Most cells in TC demonstrate atypia and are adenocarcinoma cells. A small percentage of cells in TE are adenocarcinoma cells. No atypical cells were found in other tissues (P5, P10, P15, P20, and PN). Position of sampling (including TC, TE, P5, P10, P15 and P20) were shown in the macroscopic view. PN was sampled from a distant location which is not shown in the figure. (Scale bar of microscopic view: 20 μm)

Seven tissue samples were collected from each patient after resection (Fig. 1; see “Methods” section for details). DNA methylation libraries of a total of 105 tissue samples were sequenced and analyzed.

We produced an average of 37.6 million high-quality reads for each sample; each library had over 500,000 CpG sites that had a sequencing depth no smaller than 10 (10× CpG), and over 15,000 CpG islands that had a sequencing depth no smaller than 10 (10× CGI). Our data showed a successful and consistent conversion rate (average 99.3%) of unmethylated cytosines to uracil. These allowed the sufficient genomic regions to be screened for DNA methylation markers with high confidence.

### DNA methylation markers in tumor and non-tumor tissues

**Fig. 2:**
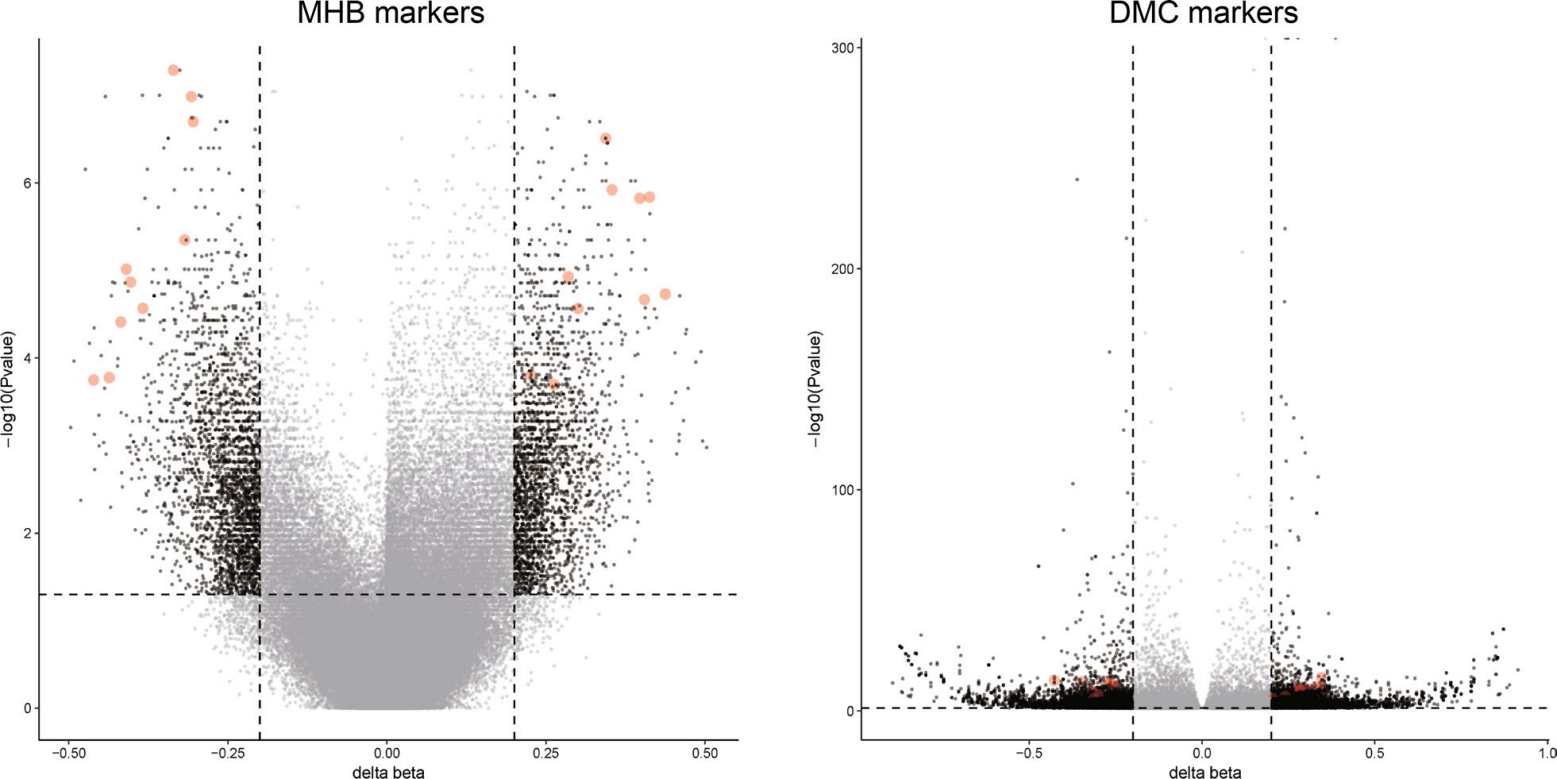
Volcano plots showing changes of the DNA methylation levels. DNA methylation levels of MHB and DMC markers between TC and PN samples are shown in volcano plots. Vertical dotted lines indicate the cutoff of the differences between TC and PN samples, and horizontal dotted lines indicate the significant cutoff. The circled dots presented the selected 20 markers in each of the MHB and DMC markers

Markers were classified as high-score markers if *M*_TC_ > *M*_PN_ and low-score markers if *M*_TC_ < *M*_PN_. In total, we identified 2284 methylation haplotype blocks (MHBs) markers (1121 high-score MHBs and 1163 low-score MHBs), 1657 differentially methylated CpG sites (DMC) markers (890 high-score DMCs and 767 low-score DMCs), and 713 differentially methylated regions (DMRs) (386 high-score DMRs and 327 low-score DMRs) (Table 2 and Fig. 2).

**Table 2:** MHB, DMC, and DMR markers identified differentially between TC and PN samples

	Total	Steep markers	Gradual markers
MHB markers	2284	1907 (83.5%)	377 (16.5%)
High-score	1121	963 (85.9%)	158 (14.1%)
AMF	501	448	53
MHL	255	218	37
MHL3	151	124	27
UMHL	116	97	19
UMHL3	84	66	18
PDR	14	10	4
Low-score	1163	944 (81.2%)	219 (18.8%)
AMF	151	127	24
MHL	76	54	22
MHL3	49	35	14
UMHL	491	406	85
UMHL3	392	319	73
PDR	4	3	1
DMC markers	234	203 (86.8%)	31 (13.2%)
High-score	172	150 (87.2%)	22 (12.8%)
Low-score	62	53 (85.5%)	9 (14.5%)
DMR markers	2085	1747 (83.8%)	338 (16.2%)
High-score	1177	996 (84.6%)	181 (15.4%)
Low-score	908	751 (82.7%)	157 (17.3%)

**Fig. 3:**
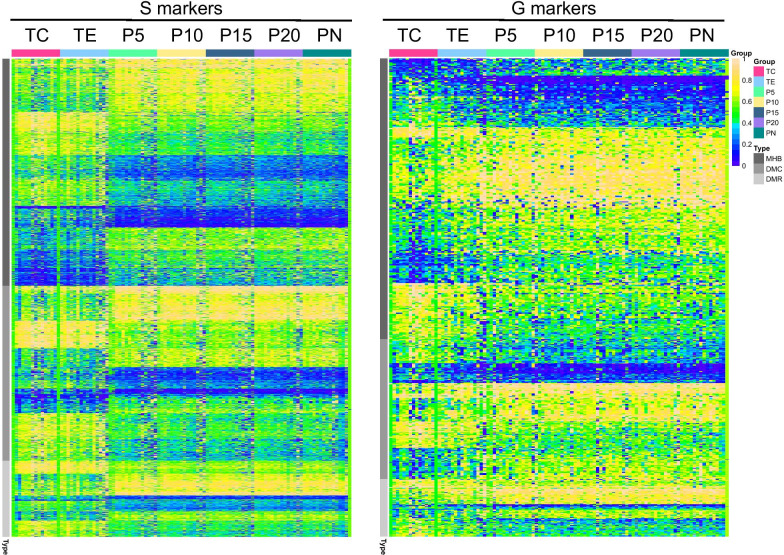
Heatmaps of identified DNA methylation markers across tumor and para-tumor. Horizontal axis indicates different markers, and vertical axis indicates different samples. S markers show a clear change between tumor samples (TC and TE) and para-tumor samples (P5–PN). G markers demonstrate a gradual change from TC to TE and to para-tumor samples (P5–PN)

From the heatmap, we observed clear trends of high-score and low-score markers, with or without an explicit conversion between TC, TE, and para-tumor tissues (Fig. 3). The methylation scores of all the high-score markers decreased in the non-tumor samples (P5–PN) compared to the tumors (TC and TE), while the low-score markers increased in the non-tumor samples compared to the tumors.

### DNA methylation markers showed different dynamic patterns from tumor to adjacent tissues

According to their dynamic changes between tumor and para-tumor tissues, we aimed to analyze the identified markers based on their trends of variations between tumor and para-tumors. To this end, we devised a metric *K*, which was defined as *k*1/*k*2 (*k*1: the difference of mean methylation scores of a given marker between TC and TE samples; *k*2: the difference of mean methylation scores of the same marker between TE and P5 samples). Based on the trends of DNA methylation scores from tumor core to para-tumor tissues, all the identified markers were classified into two subgroups according to their *K* scores. Those with a *K* score < 1 were named “Steep” (S) markers, and those with a *K* score ≥ 1 were named “Gradual” (G) markers (Table 2).

**Fig. 4:**
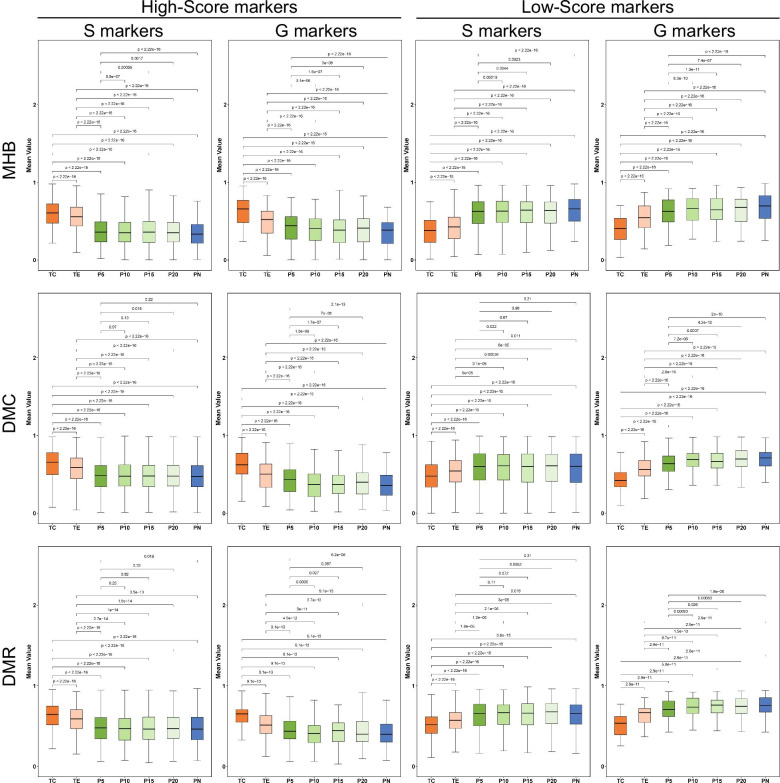
Trends of average DNA methylation levels of MHB, DMC, and DMR markers across tumor and para-tumor tissues. Each type of methylation markers was divided into four groups: high-score S markers, low-score S markers, high-score G markers, and low-score G markers. Significant difference was observed in all groups of methylation markers between TC and all other samples and between TE and all other samples

**Fig. 5:**
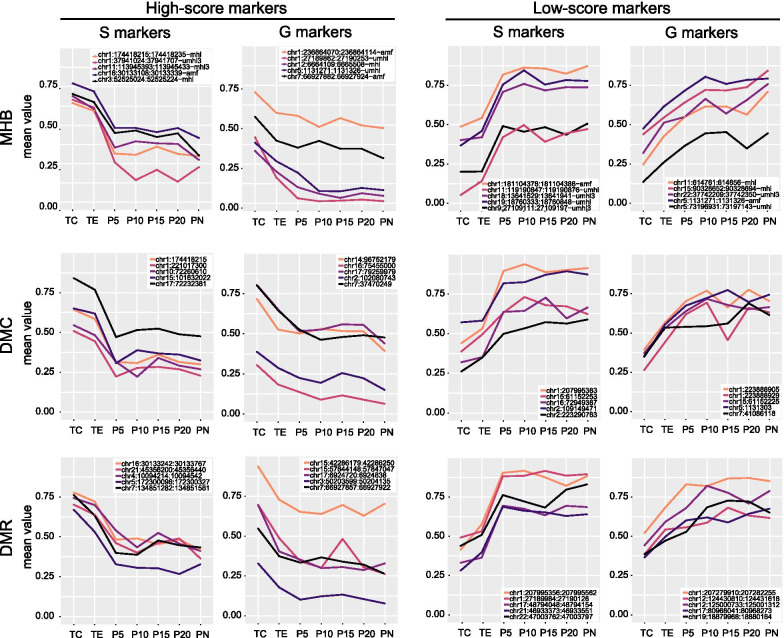
Variations of DNA methylation levels for the MHB, DMC, and DMR markers from tumor core (TC) to peripheral normal tissues (PN). Each type of methylation markers was divided into four groups: high-score S markers, low-score S markers, high-score G markers, and low-score G markers. Five representative markers were selected from each group to show their variation patterns from TC to PN. S markers showed less change in the methylation score between TC and TE than that between TE and para-tumor tissues (P5–PN) in individual samples. G markers demonstrated similar to or even greater difference between the methylation scores of TC and TE than that between P5 and TE

86.2% of the identified markers (1907 MHBs, 1469 DMCs, and 636 DMRs) were S markers. The levels of these markers changed more sharply between TE and P5 tissues than between TC and TE tissues (Figs. 3, 4), mimicking the histological change between these tissues. The mean values of all S markers of MHBs and high-score S markers of DMRs are significantly different between TC, TE, P5 and PN (FDR *p* < 0.05). The mean values of DMC S markers and DMR low-score S markers are significantly different between TC, TE, and PN (FDR *p* < 0.05). Additionally, we randomly selected 20 markers from either MHB, DMC, or DMR markers with typical variation trends to illustrate the changes in methylation levels from TC to para-tumor tissues (Fig. 5). No significant difference was observed between PN and para-tumor tissues further than P5. Some of the S markers, such as chr9:27109111:27109197 (MHB type marker, umhl3 subtype), chr2:109149471 and chr15:61152253 (DMC type), and chr4:10094214:10094542 (DMR type), had about the same methylation values between TC and TE tissues (Fig. 5).

The other markers accounting for 13.7% were G markers (377 MHBs, 188 DMCs, and 77 DMRs) whose changes from TC to TE and para-tumor samples are gradual (Figs. 3, 4). For G markers, differences between TC and TE samples were not significantly different from those between TE and para-tumor samples (from P5 to PN) (*p* = 0.132). The mean values of all G markers of MHB, DMC, and DMR are significantly different between TC, TE, P5 and PN (FDR *p* < 0.05). No significant difference was observed between PN and para-tumor tissues further than P5. Markers such as chr5:1131271:1131326 (MHB type marker, UMHL subtype), chr15:90328652:90328694 (MHB type marker, MHL subtype), chr2:102070743 (DMC type), chr7:37470249 (DMC type), chr15:42286179:42286250 (DMR type), and chr12:124430810:124431618 (DMR type) showed smaller differences in DNA methylation levels between P5 and TE than those between P5 and PN. Because these markers were explicitly prominent in TC and only modestly decreased in the P5 tissues, their presence in P5 strongly suggested that P5 might contain aberrant DNA methylations indicative of lung malignancy (Fig. 5).

### Gene Ontology (GO) enrichment of DNA methylation markers

GO analyses were conducted separately in S markers, G markers, and in high-score and low-score subgroups. GO analysis showed 281 terms that were enriched using the identified DNA methylation markers (adjusted *p* value < 0.05), including all the MHB, DMC, and DMR markers. Among them, 65 terms were shared by both S and G markers, 108 were specifically enriched in the S markers, and the rest 108 terms were only enriched in the G markers.

In KEGG pathway analysis, we found that most of the terms were closely related to cancer in the KEGG terms enriched in the S markers or subgroups. Terms such as “pathways in cancer,” “proteoglycans in cancer,” and “thyroid cancer” were directly related to cancer. The KEGG terms enriched in G markers or subgroups included: “signaling pathways regulating pluripotency of stem cells,” “transcriptional misregulation in cancer,” and “non-small cell lung cancer.”

## Discussion

In the present study, we determined the DNA methylation patterns in the tumor core and tissues at and beyond the histological margin from 15 pGGO individuals using reduced representation bisulfite sequencing (RRBS). RRBS enabled us to simultaneously access a large number of DNA methylation markers and to interrogate at CpG resolution to obtain cancer-specific methylation patterns with increased sensitivity and specificity [21, 22]. A total of 4654 cancer-specific methylation markers were selected by comparing methylation scores between tumor tissues and distal normal tissues accordingly. According to the variation patterns in the para-tumor region, these markers were further classified by our definition into S markers and G markers. S markers composed 86.2% of the tumor-related methylation markers, and G markers composed the other 13.8% percent. The methylation scores of S markers changed steeply and G markers changed gradually between the histological margin of the tumor and the para-tumor regions. S-marker-associated genes were enriched in GO terms that were related to the hallmarks of cancer. We suspected that these markers were indicative of cancerous cells, which were the most enriched in tumor core, and were still present in tumor edges. However, they were much rare, even nonexistent, in tissues beyond the histological margin. G-markers-associated genes were enriched in GO terms of stem cell pluripotency and transcriptional misregulation in cancer. These genes played a critical role in promoting tumor growth [23, 24].

Accumulating evidence supporting the theory of field cancerization established that cumulative genetic and epigenetic alterations existed in histologically normal premalignant lesions of cancer [16, 25–30]. The results of our study added to the evidence in the field of pGGO. Genetically, the same driver mutations, as identified in the cancerous tissue, were found in the nearest histologically normal tissue, P5, in two patients. Such result accorded with a previous study demonstrating that *EGFR* mutations identical to the tumors were detected in the normal respiratory epithelium in 9 of 21 (43%) patients with EGFR mutant adenocarcinomas [26]. Epigenetically, most of the S and G markers identified were found to be significantly differently methylated between P5 and PN (*p* < 0.05). Thus, subjective histological examination by pathologists might be inadequate in defining radical excision of the cancerous and precancerous tissues. And molecular pathology may assist in a more sensitive and accurate definition of surgical margin, which affects postoperative recurrence and prognosis [31].

Over the years, the resection range for GGO has been reducing from lobectomy to segmentectomy [8, 32, 33], with equivalent efficacy and safety [34–37]. However, for the utmost preservation of lung function and best prognosis, surgeons are still researching to find out the minimum margin with the lowest recurrent rate. An extremity of surgical margin was repeatedly approached by surgeons and pathologists in the spectrum of early-stage peripheral lung cancer. Wedge resection has a smaller resection range than segmentectomy, but its efficacy has been questioned [38]. A prospective study of pathological inspection recommended a safe margin of 20 mm [13]. Retrospective cohort studies found that margin distance is associated with survival. Longer recurrence-free survival (RFS) was observed in surgical margin larger than 9 mm, and longer overall survival was observed in surgical margin larger than 11 mm [14]. A single-arm prospective study named JCOG0804 demonstrated a 5-year RFS of 99.7% of sublobar resection for early-stage lung cancers smaller than 2 cm and consolidation tumor ratio (CTR) less than 0.25 [35]. The inclusion criteria required a macroscopic surgical margin of more than 5 mm. And the median macroscopic surgical margin was 15 mm. A reason of the good prognosis was that the sublobar resection removed all the cancerous and precancerous tissues [31]. In our study, methylation status was not significantly different among P10, P15, P20, and PN. And we found that genetic alterations were not identified in P10 and beyond, neither. However, genetic and methylation alterations were identified at P5 and within. Thus, we implied that surgical resection with a macroscopic margin of a minimum of 10 mm is sufficient to remove precancerous tissue with aberrant DNA methylation.

The invasive early-stage lung adenocarcinoma radiologically manifested as pGGO also served as an excellent model for surgical margin analysis. These pGGOs are presented with radiological homogeneity [39, 40] and are considered histologically and genetically homogeneous. Such intra-tumoral homogeneity paves the way for the differential analysis on methylation between tumor and normal tissues. And because invasive lung adenocarcinoma manifested as pGGO grows typically slowly, gradually invades surrounding normal tissue and changes the tumor microenvironment [41], it is plausible to assume that the landscape of precancerous methylation alteration can help understand early event in tumor progression. Thus, pGGO serves as an ideal sample for preliminary study on tumor invasion and tumor-adjacent precancerous region.

A limitation of our study is that it only included pGGO that were pathologically diagnosed as invasive adenocarcinoma. Further analysis should include GGOs with solid compartments, which are regarded as radiologically more invasive than pGGO [7]. We have already started collecting surgical samples for such a study. Comparisons should be made between pGGOs and GGOs with solid compartments in further investigation. Another limitation is that the small sample size may not support a strong level of evidence of its clinical application. However, Suzuki et al*.* have already demonstrated strong clinical evidence of the limited resection efficacy with a large cohort and a prospective study design. Larger sample size and further validation are plausible in the study of intrinsic mechanisms. Further survival analysis is also needed, since the cohort in our study adopted lobectomy and cannot provide survival evidence on limited resection with margin less than 10 mm.

## Conclusions

In the current study, we demonstrated the pattern of DNA methylation of the tumor, the tumor’s histological margin, and the para-cancerous tissues. Two variation patterns of DNA methylation levels for these markers were observed and categorized into S and G markers, aggregated on different biological pathways. We implied that surgical resection with a macroscopic margin of no less than 10 mm was sufficient to remove precancerous tissue with aberrant DNA methylation. The inclusion of epigenetic characteristics into surgical margin analysis may yield a more sensitive and accurate assessment of remnant cancerous and precancerous cells in the surgical margins.

## Methods

### Patients and sampling

Fifteen patients diagnosed as early-stage LADC with CT features of pGGO were enrolled. CT features and measures were filed by two radiologists in a back-to-back manner. All patients received a regular antibiotic treatment before the diagnosis of early-stage lung cancer. Pathology reports and staging were filed and reviewed by a certified specialized thoracic cancer pathologist. All patients enrolled had provided written informed consent, and the study was approved by the institutional review boards of Beijing Union Medical College Hospital (No. ZS-1329).

Tumor tissues in the tumor core (TC), macroscopic tumor edge, which is also microscopically confirmed as histological tumor margin (TE), para-tumor tissues at the 5 mm (P5), 10 mm (P10), 15 mm (P15), 20 mm (P20) beyond the tumor, and peripheral normal tissue (PN) were sampled separately for each patient enrolled (Fig. 1). All the samples were stored in formaldehyde within 30 min after resection. They were later prepared into formalin-fixed and paraffin-embedded (FFPE) tissues.

### DNA extraction and methylation sequencing

DNA of these FFPE samples was extracted using the QIAamp DNA FFPE Tissue Kit (Qiagen, Hilden, Germany) following the manufacturer’s instructions. All DNA methylation profiles were mapped by a reduced representation bisulfite sequencing (RRBS) protocol: Briefly, unmethylated cytosines in 200 ng input DNA was converted to uracils using the Methylcode Bisulfite Conversion Kit (ThermoFisher, MECOV50). The converted DNA samples were dephosphorylated and ligated to a universal adapter and then amplified by PCR to add indexes. The libraries were sequenced on an Illumina Hiseq X10 platform.

### Methylation markers identification and analysis

Reads were demultiplexed using the Illumina bcl2fastq software (https://support.illumina.com/sequencing/sequencing_software/bcl2fastq-conversion-software.html). For each sample, paired-end read FASTQ files were merged into single reads using PEAR (https://sco.h-its.org/exelixis/web/software/pear/doc.html). Reads in the merged FASTQ file were then adapter-trimmed using trim_galore (https://www.bioinformatics.babraham.ac.uk/projects/trim_galore/). The trimmed reads were then aligned to the bisulfite-converted human reference genome (version hg19) using Bismark (https://www.bioinformatics.babraham.ac.uk/projects/bismark/) and Bowtie2 (http://bowtie-bio.sourceforge.net/bowtie2/index.shtml).

The methylation levels of MHBs consisted of proportion of discordant reads (PDR), average methylation fraction (AMF), and methylated haplotype load (MHL) were calculated according to previous studies [42, 43]. Additional metrics including unmethylated haplotype load (UMHL), methylated haplotype load with the third power of length as the length weight (MHL3), and unmethylated haplotype load with the third power of length as the length weight (UMHL3) were also calculated for each MHB. DMC were called by R package DSS (version 2.38.0) [44]. At least three CpG sites required for one DMR and the percentage of CpG sites with significant p value (here set to 0.05) must be greater than 10%. There was no specific base pairs length defining DMR. The final DMC and DMR were and determined using the following standard: FDR < 0.05 and a mean delta-beta (Δ*β*) larger than 0.2 or less than − 0.2.

### DNA variation sequencing

For the identification of DNA variation, targeted next generation sequencing (NGS) was performed using the Next-seq500 sequencer (Illumina, Inc., CA, USA) according to the manufacturer's instruction. Samples were sequenced using a custom-designed panel of 13 lung cancer-related genes. The panel covered all the exons of *EGFR*, *BRAF*, *ERBB2*, *ALK*, *RET*, *ROS1*, *MET*, *KRAS*, *NRAS*, *PTEN*, *PIK3CA*, *TP53*, and *RET* to detect any mutations. Additionally, this panel also identifies gene rearrangements of RET, ROS1, and ALK.

### Statistical analysis

Wilcoxon rank-sum test was used to compare the status of DNA methylation markers among TC, TE, P5-20, and PN samples, followed by multiple test correction using the Benjamini–Hochberg (FDR) method. Heatmaps and boxplots were drawn by R package [45] based on the methylation levels of selected markers. Methylation markers were mapped to associated genes by the GREAT tool with default parameters (http://great.stanford.edu/) [46]. We conducted the KEGG pathway and Gene Ontology (GO) enrichment analyses using R package clusterProfiler (version 3.10.1) by default parameters except that minimal size of genes annotated by Ontology term for testing was two. Background genes were those RRBS involved. Genome protein-coding genes were used as the reference gene set for enrichment analysis. The Benjamini–Hochberg method was used for multiple testing adjustment. FDR *p* < 0.05 is considered as statistically significant.

## Supplementary Information

**Additional file 1. Supplementary Table**. Driver gene mutation and mutation-allele frequency in tumor core, tumor edge, and para-tumor tissues.** Supplementary Figure**. DNA variation of the tissue from TC, TE, P5, P10, P15, P20 and PN samples.

## Data Availability

The dataset analyzed during the current study is available from the corresponding author on reasonable request. Data generated during this study are included in this published article.

## Abbreviations

GGO: Ground-glass opacity
pGGO: Pure ground-glass opacity
MHB: Methylation haplotype blocks
DMC: Differentially methylated CpG sites
DMR: Differentially methylated regions
RRBS: Reduced representation bisulfite sequencing
S marker: Steep marker
G marker: Gradual marker
LDCT: Low-dose computed tomography
HRCT: High-resolution computed tomography
LADC: Lung adenocarcinoma
TC: Tumor core
TE: Tumor edge
P5, P10, P15, P20: Para-tumor tissues at 5 mm, 10 mm, 15 mm, 20 mm
PN: Peripheral normal tissue
MS-HRM: Methylation-sensitive high-resolution melting
IHC: Immunohistochemical
FFPE: Formalin-fixed and paraffin-embedded
