# Supplementary material for: DNA methylation patterns at and beyond the histological margin of early-stage invasive lung adenocarcinoma radiologically manifested as pure ground-glass opacity
Source: Clin Epigenetics. 2021 Aug 19;13:153. doi: 10.1186/s13148-021-01140-3 (PMC8373430; doi:10.1186/s13148-021-01140-3)

**Supplementary Online Content**

**Jia et al. DNA methylation patterns at and beyond the histological margin of early-stage invasive lung adenocarcinoma radiologically manifested as pure ground-glass opacity**

[Supplementary Table 1. Driver gene mutation and mutation-allele frequency in tumor core, tumor edge, and para-tumor tissues 2](#_Toc76158615)

[Supplementary Figure 1. DNA variation of the tissue from TC, TE, P5, P10, P15, P20 and PN samples. 3](#_Toc76158616)

This supplementary material has been provided by the authors to give readers additional information about their work.

## Supplementary Table 1. Driver gene mutation and mutation-allele frequency in tumor core, tumor edge, and para-tumor tissues

| ID | Gene status | Mutation-allele frequency | | | | | | |
| --- | --- | --- | --- | --- | --- | --- | --- | --- |
|  |  | TC | TE | P5 | P10 | P15 | P20 | PN |
| 1 | *EGFR* L858R | 0.064 | 0.089 | 0 | 0 | 0 | 0 | 0 |
| 2 | *EGFR* G719A | 0.079 | 0.026 | 0 | 0 | 0 | 0 | 0 |
| 3 | *EGFR* L858R | 0.140 | 0.118 | 0 | 0 | 0 | 0 | 0 |
| 4 | *EGFR* L858R | 0.470 | 0.445 | 0.012 | 0 | 0 | 0 | 0 |
| 5 | *EGFR* L858R | 0.083 | 0.214 | 0.010 | 0 | 0 | 0 | 0 |
|  | *PTEN* C136Y | 0.006 | 0 | 0 | 0 | 0 | 0 | 0 |
| 6 | Wild type | WT | WT | WT | WT | WT | WT | WT |
| 7 | *EGFR* L858R | 0.256 | 0.174 | 0 | 0 | 0 | 0 | 0 |
| 8 | *EGFR* exon19del | 0.113 | 0.203 | 0 | 0 | 0 | 0 | 0 |
| 9 | *EGFR* L858R | 0.102 | 0.204 | 0 | 0 | 0 | 0 | 0 |
| 10 | *EML4*-*ALK* fusion | 0.009 | NA | NA | NA | NA | NA | 0 |
| 11 | Wild type | WT | WT | WT | WT | WT | WT | WT |
| 12 | Not available | NA | NA | NA | NA | NA | NA | NA |
| 13 | *ERBB2* exon20 ins | 0.046 | 0.120 | 0 | 0 | 0 | 0 | 0 |
| 14 | Wild type | WT | WT | WT | WT | WT | WT | WT |
| 15 | EGFR exon19del | NA | NA | NA | NA | NA | NA | NA |

*WT: wild type, NA: not available, TC: tumor core, TE: tumor edge, PN: peripheral distal normal tissue

## Supplementary Figure 1. DNA variation of the tissue from TC, TE, P5, P10, P15, P20 and PN samples.

DNA variation of nine patients with sufficient tissue were assessed. ‘row min’ and ‘row max’ indicated the minimal MAF (mutation allele frequency) and maximal MAF of each row, respectively.


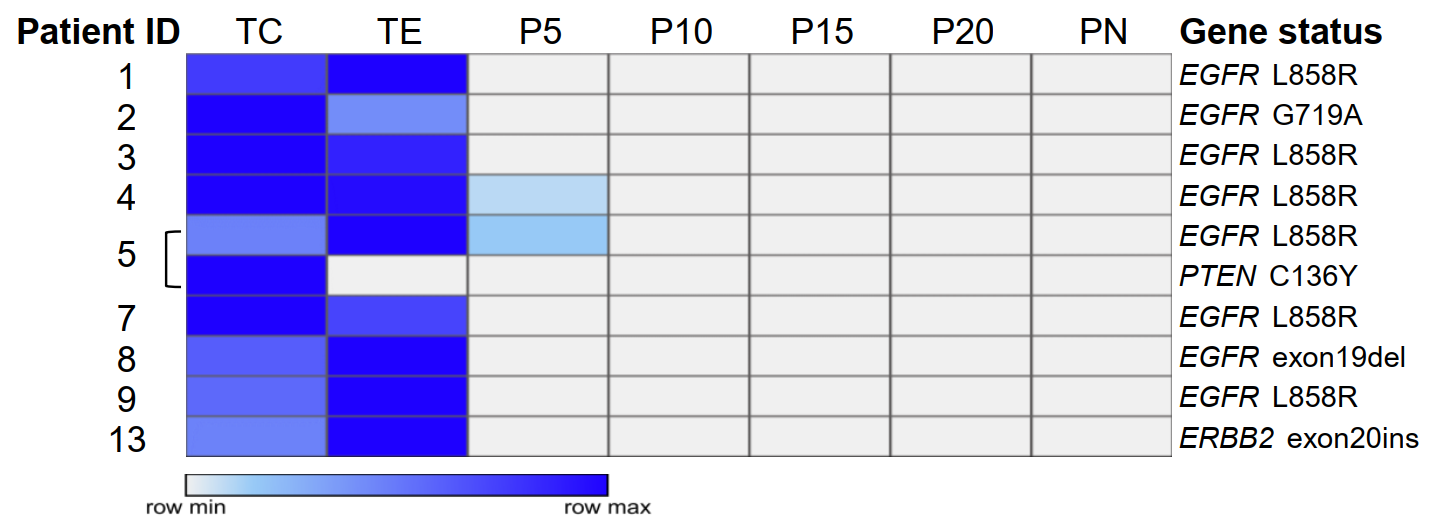

Supplement: Supplementary file 1 — Additional file 1. Supplementary Table. Driver gene mutation and mutation-allele frequency in tumor core, tumor edge, and para-tumor tissues. Supplementary Figure. DNA variation of the tissue from TC, TE, P5, P10, P15, P20 and PN samples. [file 13148_2021_1140_MOESM1_ESM.docx]
